# Job crafting and teachers’ well-being: a case of private school teachers

**DOI:** 10.1186/s12889-025-25945-6

**Published:** 2025-12-13

**Authors:** Azra Aslam, Mahwish Kamran, Sohni Siddiqui

**Affiliations:** 1 Department of Education and Social Sciences, Iqra University, Karachi, 75500 Pakistan; 2 Institute for Educational Research, University of Wuppertal, Wuppertal, Germany

**Keywords:** Job crafting, Well-being, Work life balance, Job satisfaction and burnout, Work environment, Thematic analysis

## Abstract

The well-being of teachers is a widely studied topic, often in relation to how long they stay in the profession. Despite significant efforts, retaining teachers remains a global challenge. This qualitative study examined the impact of job crafting on work-life balance, job satisfaction, and burnout among teachers in some private schools in Karachi, Pakistan, taking into account their working environment. Through thematic analysis, the research identified key practices that enabled teachers to thrive in their institutions for at least five years. These included time management, building social support networks, prioritising tasks, and engaging in professional development. These strategies helped teachers combat burnout, achieve work-life balance, and find job satisfaction. While acknowledging the study's limitations, these findings offer valuable insights to Pakistani educational institutions seeking to foster a healthy, efficient, and proactive teaching workforce.

**Supplementary Information:**

The online version contains supplementary material available at 10.1186/s12889-025-25945-6.

## Introduction

Teaching is the fundamental profession from which all others stem, and it is a profession that is both noble and important [1]. Therefore, the best minds should remain in education; however, teacher retention is one of the main issues worldwide [4, 21, 27]. Despite the honour and respect associated with this profession, many teachers leave their jobs within the first five years of their career [26]. According to the results of a five-year longitudinal study conducted by Räsänen et al. [27] in Finland and involving 2,310 high school teachers, 50% of teachers showed intention to leave their jobs for various reasons. Similar studies have been reported in the context of Pakistan. According to Javaid [12], based on the first ever data compiled by the Sindh Government on private schools in the province, approximately 3.9 million students are enrolled in private schools. For these students, 225,158 members of teaching staff are available. Of these, 30–50% reported turnover intention within the first five years of their tenure [21], due to reasons such as an excessive workload, a lack of support, non-competitive salary packages, and insufficient professional development opportunities. Siddiqui et al. [31] found that Pakistani teachers are leaving the profession due to excessive integration of technology without sufficient training. One study also reported that married teachers usually complain more about difficulty in maintaining work-life balance [25], showing that, despite teaching being one of the few professions open to women in Pakistani society, these circumstances further limit their engagement with career and professional growth [16]. Furthermore, losing a trained teacher puts a strain on the school's budget and affects students' outcomes [1]. Consequently, many schools make significant efforts to retain their trained teachers, albeit without success [4]. Much research has been conducted internationally to address the issue of retaining teachers within the profession and to support and guarantee teachers' well-being [8, 38]. However, limited research has focused on teachers' personal attributes [7].

According to Wrzesniewski and Dutton [39], job crafting is the process by which employees redefine and reimagine their roles to enhance personal meaning. Through job crafting, employees can independently modify aspects of their work to better align it with their individual needs, preferences, and capabilities [3]. Siddiq et al. [30] studied the concept of job crafting in relation to teachers’ job performance, and the results were astounding. According to these results, teachers who practice job crafting reported greater job satisfaction and organisational commitment. In addition, job crafting has already been identified as a significant factor contributing to teachers' mental wellbeing and satisfaction [6, 7, 23, 41]. According to Wrzesniewski and Dutton [39] further elaborated that there are three basic types of job crafting: task crafting which involves shaping the boundaries and processes of a job, relational crafting which involves shaping the quality and quantity of job-based relations,and cognitive crafting, which involves shaping one’s attitude towards one’s job in any given environment. This study examines the overall impact of job crafting, in relation to its different types, on important teacher outcomes such as satisfaction levels, work-life balance and burnout reduction. This study is unique in its focus on private high school teachers who deliver international curricula in a major Pakistani city.

## Literature review

### Theoretical foundation

The theoretical basis for this research comes from Cognitive Evaluation Theory (CET), a component of Self-Determination Theory (SDT). CET proposes that intrinsic motivation emerges when an individual feels their need for autonomy and competence is met [28]. Job crafting is a proactive approach in which teachers tailor their job tasks, peer relationships, and the cognitive dimensions of their work, thereby expanding their sense of autonomy and competence. Research shows that when individuals' needs for competence and autonomy are not met, they report rising levels of burnout, an imbalanced work-life relationship and declining job satisfaction [17]. Studies, particularly those conducted in educational settings, emphasise that teachers experience greater job satisfaction, a better work-life balance, and reduced burnout when offered autonomy in restructuring their roles [9, 29]. Therefore, it is clear that CET provides valuable theoretical support for the idea that job crafting positively affects teachers' well-being by enabling them to shape their jobs, reducing burnout and improving work-life balance and job satisfaction.

### Job crafting and its relation with teachers’ burnout

Burnout has emerged as a central topic of discussion in recent years due to its strong link to major stress-related illnesses [40]. Burnout is defined as a state of imbalance between job demands and available resources [35], characterised by mental and physical fatigue [19]. According to Teles et al. [34], 54% of teachers acknowledge that teaching is a highly stressful profession and 30% appear to be in a 'pre-burnout' state. However, research also suggests that job crafting can help in reducing burnout and stress [7]. According to Mushtaq and Mehmood [19], employees who are overworked but learn to adapt and craft their roles report improved well-being. Similarly, Dreer [7] acknowledged the positive impact of job crafting on teachers' burnout and well-being, as it enables them to adapt their roles to suit their preferences and abilities. Furthermore, job crafting interventions lead to greater empowerment, increased engagement at work, and reduced burnout [19]. A prominent study by Toropova et al. [35] confirmed that physical and behavioural factors in the school environment play a significant role in job satisfaction and that their absence can lead to burnout [34]. Discussion of burnout has become one of the main topics in recent years as it is considered a major cause of several stress-related illnesses [40]. According to research cited by Teles et al. [34], 54% of teachers acknowledge that teaching is a highly stressful profession and 30% were found to be in a 'pre-burnout' state, reporting utter dissatisfaction in balancing their professional and personal lives.

### Job crafting and its relation with work–life balance

Work–life balance is defined as achieving equilibrium between work and personal life [8]. As technology is increasingly used in education, the distinction between working time and family time is becoming more blurred [5]. Stephen and Elza [32] also found that mothers in particular are disproportionately affected by imbalances, as they need to spend quality time with their children during their formative years. Unsurprisingly, work and life are interrelated [14], meaning a setback or breakthrough in one area is also felt in the other. Personal worries can distract people, causing them to feel troubled and disengaged at work throughout the day. Similarly, work issues can affect personal life. Ultimately, dissatisfaction in any area of life leads to a general feeling of unhappiness [22]. Kardas [13] conducted a study investigating two forms of job crafting: task crafting, which involves modifying job activities, and cognitive crafting, which involves altering the mental perception of work. The study found a strong correlation between job crafting and work-life balance, as well as a positive association between the development of job crafting and job satisfaction. The high level of job satisfaction among the employees studied suggested that they had achieved a good work-life balance. Building on these findings, the current study specifically examines this connection among teachers in private high schools in Karachi. The current research study highlights the concept of job crafting as a possible solution for dealing with the negative impact on work–life balance.

### Job crafting and its relation with job satisfaction

According to Meier and Spector [18], job satisfaction is an individual's subjective evaluation of their job, failing which may lead to decreased productivity, burnout, and physical and mental issues. The antecedents of job satisfaction can fall under several categories such as job security [19], work–life balance, and peer support [2]. The positive impact of job crafting on job satisfaction is established by several studies [6, 23, 41]. According to Dreer [7], the sense of autonomy achieved by job crafting helps teachers achieve greater job satisfaction. It helps them feel more in control of their tasks, design their tasks, and thus feel less drained and more satisfied [7]. In continuation, Zito et al. [41] demonstrated that job crafting acts as a mediating mechanism, linking job autonomy positively to both job satisfaction and work-family conflict.

### Job crafting and work environment

A positive work environment is crucial for enhancing worker performance and motivation, as it fosters collaboration, productivity and job satisfaction. A research study by Urbanaviciute and Lazauskaite-Zabielska [36] found that job crafting can lead to a more balanced and favourable work environment, thereby increasing work engagement. Conversely, avoiding job crafting is directly linked to lower work engagement, regardless of the work environment [36]. Bakker et al. [2] further explained that employees' efforts to change their job crafting practices can positively impact their own and their colleagues' work engagement. Another study found that employees who proactively increase their job resources, such as support from colleagues or opportunities for growth, and challenge their job demands, such as taking on more responsibility, become more engaged at work [2]. This positive effect also spreads to their colleagues, as people tend to imitate each other's behaviour [2]. Importantly, studies have shown that job crafting enables teachers to navigate challenging work environments by reshaping their tasks and relationships. This helps to sustain engagement, even in contexts where resources are limited [37].

### Linking job crafting with burnout, work–life balance, and job satisfaction

A review of the literature shows that job crafting significantly impacts teachers’ well-being by influencing various aspects of their professional lives. Specifically, it reduces burnout by enabling teachers to transform overwhelming demands into manageable tasks [7], fosters work–life balance by offering flexibility and autonomy in organising responsibilities [8, 32], and enhances job satisfaction by promoting meaningful engagement and a sense of control [4]. While international research highlights these positive outcomes, there is limited empirical evidence of how teachers apply job crafting strategies in their everyday practice locally [7]. Examining the interconnections between job crafting, burnout, work–life balance and job satisfaction among teachers in Pakistan could therefore provide new insights for both theory and practice, offering pathways to enhance teacher well-being and educational quality. Job crafting is a relatively new concept in the field of education [7]. Therefore, to address this gap, it was important to study the relationship between job crafting and teachers' well-being in a local context to gain a new understanding.

## Research methodology

### Research setting

This study focused mainly on co-educational high schools in Karachi that had teachers of both genders and had been in business for at least fifteen consecutive years. This study mainly focused on teachers with five years or more of experience, working in established high schools in Karachi, which teach international curricula such as IB and CAIE. These schools anticipate rigorous academic expectations, resulting in heavier workloads and complex demands from students, management, and the parent body. Selecting experienced teachers was intentional, since they often apply self-developed strategies that reflect job crafting. This context provided a practical setting to explore how job crafting impacts job satisfaction, work-life balance, and burnout.

### Research design

For this qualitative research, 30 interviews were conducted with teachers from high schools in Karachi that have been teaching international syllabuses (such as Cambridge and IB) for more than fifteen years. The sample size of 30 participants was determined through purposive sampling, which is an appropriate method for qualitative enquiry with a focus on detailed, context-rich exploration [33]. To ensure adequate data, interviews continued until data saturation was reached, i.e. when no new themes or insights emerged from additional interviews. This approach aligns with established qualitative research standards and ensures the trustworthiness of the findings. All of the teachers interviewed had more than five years of experience (see Table 1). For this study, an interview guide was developed to explore teachers’ perceptions of their work environment, professional challenges, well-being, burnout, and job satisfaction. This consisted of open-ended questions designed to elicit in-depth responses (see Supplementary File 1: Interview Guide). The guide was reviewed by experts to ensure the relevance and clarity of its content.

**Table 1:** Participant and institution characteristics

Category	Details
Participants	Teachers (*N* = 30) Male = 10 Female = 20
Characteristics of participants selected for an interview	• Teaching experience of more than 5 years • Grades IX to XI • Teaching any subject • Attended professional development courses
Characteristics of the institutions	• Teaching international syllabi (Cambridge, IB, etc.) • More than 15 years in business

To ensure confidentiality, the names and characteristics (such as gender, experience and institution) of the respondents are not disclosed. Data analysis was carried out using open coding, in line with the methodology outlined by Strauss and Corbin [33]. Themes were then identified and presented in this study.

### Sampling characteristics

This research used a purposive sampling technique. Participants were selected based on the following criteria: having worked in the profession for at least 5 years in an educational institution that had been established for at least 15 years, and being part of a mixed-gender workforce. Eight schools were selected, with four to five teachers from each school given the opportunity to participate in the study. One-to-one, face-to-face interviews were conducted with the respondents. The interview guide was validated through an extensive literature review and feedback from four experts, including senior faculty members and practitioners in the field, to ensure authenticity and content validity. Furthermore, a brief pilot study with five participants also guaranteed that the questions permitted detailed and thoughtful responses and ensured clarity. All ethical standards, such as confidentiality, the participants' right to withdraw, and informed consent, were strictly adhered to throughout the process.

## Qualitative thematic analysis

The interviews were transcribed by the first author under the guidance of the supervisor (second author). Thematic analysis of the transcripts centered primarily on the practices leading to the concept and application of job crafting, which enables the teachers to achieve work-life balance, perceive their level of job satisfaction, and deal with burnout in their work environment. To ensure a comprehensive understanding of the data, the verbatim transcripts underwent multiple rounds of reading and revisitation. These transcripts were then systematically coded. The resulting emergent codes were subsequently grouped into relevant categories and themes, leading to the identification of final patterns that directly addressed the study's research objectives. Data analysis involved open coding, following the methodology detailed by Strauss and Corbin [33]. Subsequently, themes were identified and presented in this study. Some of the themes generated are discussed below.

### Task crafting: discipline, organization, and time management

The uniqueness of job crafting lies in its proactive nature, where individuals actively redesign their tasks to enhance their well-being [15]. In this study, many teachers emphasized discipline and organization as central to managing their responsibilities. For example, one teacher shared that she ensures her *“schedule of checking is followed well, and her priorities are set according to the work assigned to her.”*

Similarly, another teacher stated:

“*I believe in setting realistic goals. At times, I select only that task which is of utmost importance since I am a single parent and my responsibilities are multi-fold. Yet on other days, when I am more at ease mentally, I prefer completing as much work as possible, as soon as it is assigned, without waiting for the deadlines.”*

Such practices align with task crafting, where teachers classify and prioritize tasks to minimize stress and achieve balance. Effective time management enables them to reduce burnout, complete work within school hours, and reserve time for personal responsibilities. As Dreer [7] suggests, task crafting helps in categorizing tasks based on urgency and importance, thereby improving efficiency and sustaining work–life balance. Thus, task crafting in the form of time management directly links to teachers’ well-being, as it reduces burnout, enhances work-life balance, and increases satisfaction when successfully practiced.

### Cognitive crafting: professional development and growth

Professional development opportunities were seen as crucial for enhancing teachers’ self-efficacy and capacity to craft their jobs effectively. Teachers who had access to such opportunities reported greater autonomy and satisfaction, which echoes Nipper et al.’s [20] argument that professional growth equips employees to take control of their tasks while supporting organizational goals.

One participant explained, *“seeking professional development opportunities, mentorship, and support from colleagues and educational networks to stay updated on best practices and address challenges effectively.”*

Another shared, *“I try different strategies to make my teaching more interesting for learners by attending workshops, watching videos, and reading material related to professional development.”*

From the Job Demand-Resources (JD-R) model, opportunities for professional development act as that valuable resource which cushions the negative impacts of burnout, particularly in times of high job demands [11].

A teacher stated: *“As a teacher, I have learnt a lot here. I appreciate opportunities for my professional growth and development, positive work culture, supportive leadership, autonomy, and because of that, I’ve achieved a greater sense of purpose and meaning in my work”.*

Such examples reflect cognitive crafting, where teachers reframe their jobs in terms of growth, purpose, and meaning. Professional development, therefore, acted as both a personal and institutional resource that buffered burnout and strengthened job satisfaction.

### Cognitive crafting: recognition and rewards

Another key theme was the perceived sense of recognition and rewards among teachers, which is directly linked to cognitive crafting in that it shapes how individuals interpret the value of their work [39]. Recognition—both verbal and monetary—was seen as validation of their contributions and a source of motivation. As one teacher noted, *“Appreciation and a comfortable environment are two main features I like about the organization. In my organization, efforts are recognized, and ideas are appreciated. It’s one of the reasons I have been working here for more than a decade now.”*

Others highlighted tangible benefits: *“Although there is much verbal appreciation, there are also sufficient monetary benefits. Besides that, teachers are acknowledged for their exceptional board results, in the form of appreciation certificates, shields and, a promising increment next year. Everyone feels motivated to perform even better.”*

At the same time, respondents also expressed concerns about insufficient financial compensation: *“Indeed, teaching is my passion, and it’s a noble profession but, you see, here, we are giving the best years of our lives. So, just like in the corporate sector, our schools should also cover health insurance and a provident fund for their teachers.”* Another shared, *“Mere appreciation in the form of shields, certificates, and a round of applause is not enough. Something more needs to be done.”*

These reflections emphasize that recognition and rewards reinforce teachers’ sense of purpose, strengthen job satisfaction, and boost self-worth. As Żytko [42] argues, appreciation in teaching professions—where material benefits are often limited—becomes central to sustaining motivation and well-being. In job crafting terms, recognition and rewards strengthen cognitive crafting by shaping how teachers view the meaning and value of their work, which in turn nurtures well-being through motivation, self-worth, and purpose.

### Relational crafting: building supportive work-based relationships

Relational crafting was one of the most prominent strategies reported by teachers. Supportive relationships with colleagues created a sense of belonging and directly influenced their well-being. One respondent described, *“collaborative and supportive practices are very helpful.”* Another shared, *“My work-based relationships provide emotional support, collaboration opportunities, and professional networking, enhancing both personal satisfaction and career growth.”*

These accounts reflect Harju et al.’s [10] claim that relational crafting transforms workplace interactions, motivating employees to actively reshape their roles. Teachers explained how collegial support fostered motivation and resilience. For example, one participant said, *“We (work-based relationships) help each other a lot during and beyond our working hours”*.

Similarly, one other teacher stated: *“I appreciate that we have an open-door policy by the administrator for listening to and resolving issues, leaves, half days in case of emergency, medical insurance, time to sit and collaborate with colleagues, *etc*. It refreshes me within a few minutes”.*

Although relational crafting requires proactive effort, it was clear that supportive networks helped teachers reduce burnout, manage work–life balance, and experience greater job satisfaction.

### Job crafting and teachers’ well-being in private schools

Findings from the study highlight how job crafting—through task, cognitive, and relational strategies—directly contributed to teachers’ well-being. Task crafting enabled them to manage time and responsibilities effectively. Cognitive crafting, achieved through professional development, rewards, and recognition, provides them with autonomy and purpose. Relational crafting fostered supportive networks that reduced burnout. Teachers in private schools described how these strategies transformed their workplace into more than just a site of labour. As one respondent shared, *“It is because of the wonderful people at work that I feel like never leaving my organization.”* Over time, these practices created a sense of community, enhanced job satisfaction, and helped sustain both personal and professional well-being.

## Discussion

This study examined how teachers in private schools engage in job crafting to manage burnout, balance personal and professional responsibilities, and maintain job satisfaction. Using Wrzesniewski and Dutton’s [39] framework of task, cognitive and relational crafting as a basis, the findings show that teachers proactively reshape their roles to enhance well-being, autonomy and engagement. Recognition and rewards emerged as an additional dimension of cognitive crafting, suggesting that existing models should be refined to capture contextual factors in education.

In line with previous studies [19], participants reported high stress levels due to heavy workloads and limited resources. However, their proactive strategies, such as reorganising schedules, prioritising tasks and redefining goals, demonstrate the agency inherent in job crafting. These findings reinforce the theoretical proposition that job crafting reduces burnout by restoring a sense of control, while also highlighting its limitations. Systemic factors such as heavy teaching loads and inadequate institutional support require organisational interventions alongside individual strategies.

Work–life balance emerged as a significant concern, particularly among women with caregiving responsibilities. Teachers were able to better integrate their personal and professional domains through task crafting (e.g. adjusting teaching commitments) and cognitive reframing (e.g. redefining boundaries between work and home). This aligns with previous research indicating that job crafting can mitigate negative spillover between life domains [22]. However, the persistence of imbalance highlights the practical necessity for schools to implement family-friendly policies and structural support, as individual efforts alone are insufficient.

The study also emphasises how job crafting can increase job satisfaction. Key contributors were autonomy, meaningful work, collaborative relationships and recognition. The emergence of recognition as a critical aspect of cognitive crafting extends theoretical frameworks by illustrating how intrinsic motivations and extrinsic incentives both shape engagement and well-being. Relational crafting was also found to be essential in sustaining supportive work environments, which is consistent with the findings of Harju et al. [10]. However, the effectiveness of these strategies is constrained by structural limitations such as inadequate health insurance.

This study makes a unique contribution to the literature by explaining job crafting in the context of a South Asian private school, demonstrating both the applicability of existing frameworks and the need for contextual extensions. Identifying recognition and rewards as a distinct sub-dimension of cognitive crafting is particularly relevant in undervalued professions and low-resource settings. In practice, the findings emphasise the importance of additional institutional measures, such as professional development, workload management, and financial and non-financial recognition, to maximise the benefits of individual job crafting strategies. Integrating individual and organisational perspectives strengthens the theoretical and practical relevance of the study.

The summary of the themes and sub themes emerged are presented in Table 2.

**Table 2:** Themes and overarching concepts of each individual theme

Themes	Sub-Themes and Practices
Time Management and prioritizing tasks	• Creating to-do lists • Goal setting • Scheduling tasks • Meeting deadlines
Perceived sense of Rewards and Recognition	• Acknowledgement of efforts • Intrinsic motivation • Extrinsic rewards
Building Social Support Network	• Emotional support • Communication & collaboration • Reduced feelings of work-related stress • Motivation & fulfillment
Engaging in Professional Development Activities	• Motivation • Collaborative practices • Autonomy & resourcefulness • Sense of purpose

## Conclusion

Overall, this study demonstrates that job crafting is an effective way for teachers in Pakistan to manage burnout, achieve work–life balance, and increase job satisfaction. However, its full potential can only be realised when individual agency is coupled with institutional reforms. Schools can enhance teacher well-being and educational quality by investing in supportive environments and recognising teachers’ contributions. Furthermore, the study produced some key findings. Firstly, the study revealed that, although teachers are actively reshaping their roles and tasks to sustain their well-being, recognition and rewards have emerged as an additional aspect of cognitive crafting. This highlights the importance of both intrinsic and extrinsic motivation. Secondly, work-life balance remained a major concern, with a focus on relational crafting. Thirdly, in a low-resource context such as ours, combining teachers' individual strategies and institutional support has been observed to be the most sustainable way to promote teacher well-being.

## Limitations, strengths and direction for future research

The scope of this study is limited by its focus on private schools offering an international curriculum, as well as by its qualitative design, which restricts its generalisability. Our study targeted teachers in private schools offering international curricula in Pakistan because these syllabuses are only taught in the private sector. Teachers from private international schools were selected due to the increasing pressure on these institutions to prepare students for the job market and university admissions, particularly at an international level. Consequently, the workload in these schools is typically higher than in public schools, which is why they were the primary focus of our research. Another limitation is that well-being is a broad concept and many of its aspects have been overlooked. Future research should employ mixed methods or large-scale quantitative approaches to test causal links between job crafting and well-being. Comparative studies between the public and private sectors, as well as gender-sensitive analyses, would further enrich our understanding of job crafting in diverse educational settings.

It is important to note that our study focused specifically on participants with an established professional identity and sufficient autonomy, prerequisites for engaging meaningfully in job crafting behaviours. Novice teachers, who are often focused on adapting to institutional expectations and mastering classroom management, may lack the confidence or opportunity to engage in such proactive role modification. However, comparing the job crafting practices of novice and experienced teachers could be a compelling focus for a separate future study.

Despite these limitations, this study is one of the few to examine the selected themes together. Job crafting is an understudied phenomenon which has mostly been studied in university settings. This study attempted to examine this phenomenon among private high school teachers.

## Recommendation

In times of high economic demand, few people pursue a career in teaching [24]. Therefore, it is crucial that teachers are trained in job crafting practices to help them adapt while maintaining their well-being. According to Cells et al. [4], taking pre-emptive steps such as creating an open, democratic and sociable environment fosters an atmosphere conducive to learning and promotes the psychological health of teachers.

## Supplementary Information

Supplementary Material 1.

## Data Availability

The data supporting the findings of this study are available from the corresponding author upon reasonable request.
