# Supplementary material for: Job crafting and teachers’ well-being: a case of private school teachers
Source: BMC Public Health. 2025 Dec 13;26:239. doi: 10.1186/s12889-025-25945-6 (PMC12821878; doi:10.1186/s12889-025-25945-6)
Supplement: Supplementary file 1 — Supplementary Material 1. [file 12889_2025_25945_MOESM1_ESM.docx]

**Interview Guide (English Version for Supplementary File)**

**Introduction (for participants)**

- Thank you for agreeing to participate.
- This interview explores your experiences with work-related challenges, organizational culture, and well-being.
- Your responses will remain confidential and used only for research purposes.
- There are no right or wrong answers; please share your honest views.

**Interview Questions**

1. What role does technology play in helping you manage professional challenges in your work?
2. In what ways do your workplace relationships (e.g., colleagues, supervisors) support your personal and professional well-being?
3. What areas do you think your organization needs to improve to better support teachers?
4. How are professional achievements usually recognized or celebrated in your organization? Do you think this is sufficient, or could it be more effective?
5. What is the company/school culture or policy regarding the prioritization of employees’ well-being?
6. How easy is it for you to mentally “switch off” from work during your time off?
7. In your opinion, how does burnout affect the quality of one’s personal and professional life?
8. Which school policies, in your view, particularly contribute to burnout among teachers?
9. What aspects of your professional experience in your current organization do you appreciate the most?
10. What suggestions would you like to share for improving job satisfaction among teachers in general?
